# ChEAP: ChIP-exo analysis pipeline and the investigation of *Escherichia coli* RpoN protein-DNA interactions

**DOI:** 10.1016/j.csbj.2022.11.053

**Published:** 2022-12-02

**Authors:** Ina Bang, Linh Khanh Nong, Joon Young Park, Hoa Thi Le, Sang- Mok Lee, Donghyuk Kim

**Affiliations:** a School of Energy and Chemical Engineering, Ulsan National Institute of Science and Technology (UNIST), Ulsan 44919, Republic of Korea; b Schools of Life Sciences, Ulsan National Institute of Science and Technology (UNIST), Ulsan 44919, Republic of Korea

**Keywords:** ChIP, Chromatin Immunoprecipitation, Colab, Google Colaboratory, ChEAP, ChIP-exo Analysis Pipeline, TRN, Transcriptional Regulatory Network, TF, Transcription Factors, SNR, Signal-to-Noise Ratio, ChIP-exo, Analysis pipeline, RpoN, Sigmulon, Sigma factor, *Escherichia coli*

## Abstract

Genome-scale studies of the bacterial regulatory network have been leveraged by declining sequencing cost and advances in ChIP (chromatin immunoprecipitation) methods. Of which, ChIP-exo has proven competent with its near-single base-pair resolution. While several algorithms and programs have been developed for different analytical steps in ChIP-exo data processing, there is a lack of effort in incorporating them into a convenient bioinformatics pipeline that is intuitive and publicly available. In this paper, we developed ChIP-exo Analysis Pipeline (ChEAP) that executes the one-step process, starting from trimming and aligning raw sequencing reads to visualization of ChIP-exo results. The pipeline was implemented on the interactive web-based Python development environment – Jupyter Notebook, which is compatible with the Google Colab cloud platform to facilitate the sharing of codes and collaboration among researchers. Additionally, users could exploit the free GPU and CPU resources allocated by Colab to carry out computing tasks regardless of the performance of their local machines. The utility of ChEAP was demonstrated with the ChIP-exo datasets of RpoN sigma factor in *E. coli* K-12 MG1655. To analyze two raw data files, ChEAP runtime was 2 min and 25 s. Subsequent analyses identified 113 RpoN binding sites showing a conserved RpoN binding pattern in the motif search. ChEAP application in ChIP-exo data analysis is extensive and flexible for the parallel processing of data from various organisms.

## Graphical abstract

**Figure:**
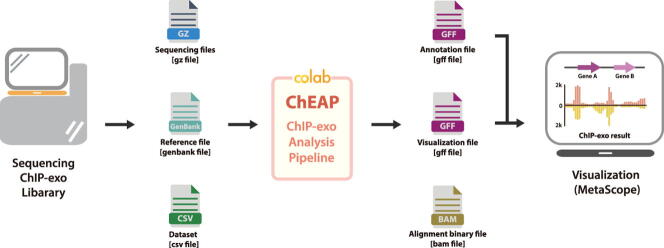

## 1 Introduction

**Fig. 1:**
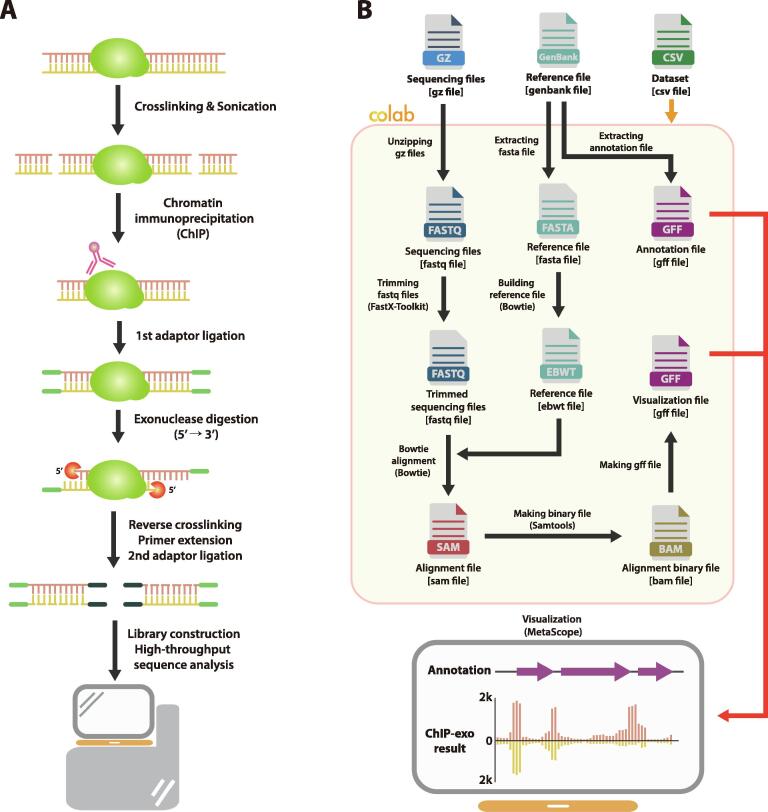
Schematic diagram of ChIP-exo experiment and its processing with ChEAP. (A) ChIP-exo experiment typically starts with cross-linking of DNA-binding proteins and fragmentation of DNA-protein complexes with sonication, followed by the selective enrichment for target protein using a specific antibody. Exonuclease treatment digests the unbound single DNA from the 5′ → 3′ end direction. Reverse cross-linking, second strand synthesis, adaptor ligation, and library construction are performed subsequently. Sequencing of libraries results in ChIP-exo sequence reads in the FASTQ format. (B) ChEAP analysis workflow begins with three input files which include a raw ChIP-exo sequence reads file, a reference genome file, and a summary sheet of the dataset in CSV format. Input files can be uploaded to the user’s Google Drive when ChEAP is executed in the Google Colab environment. Names of bioinformatic tools used at each step of ChEAP are indicated with round parentheses. MetaScope can visualize GFF files of ChIP-exo results along with the genome annotation (red arrow).

Genome-wide reconstruction of the transcriptional regulatory network (TRN) in bacteria is enabled by the development of chromatin immunoprecipitation (ChIP) methods. ChIP techniques use formaldehyde to stabilize proteins at their *in vivo* binding locations. The subsequent addition of specific antibodies selectively enrich for DNA-binding proteins of interest and their associated DNA fragments thus, precisely map the binding sites to identify their regulatory targets. In bacterial studies, ChIP followed by microarray hybridization (ChIP-chip), or high-throughput sequencing (ChIP-seq) have proven their utilities in defining regulons, and binding patterns of several TFs under different growth conditions [1], [2], [3], [4], [5]. ChIP with λ exonuclease digestion followed by sequencing (ChIP-exo) (Fig. 1A) was developed to further improve the signal-to-noise ratio (SNR) and sharpen the binding peaks to a near-single base pair (bp) resolution. ChIP-exo allows the detection of smaller peaks, and distinguishes clustered binding events, that are often difficult with ChIP-seq [6]. In bacterial TRN investigation, ChIP-exo has been adopted to reveal the extensive interaction of the transcription factors (TFs) beyond their general regulatory roles [7], [8], [9], [10].

As the cost of next generation sequencing (NGS) continues to decline, ChIP-seq and ChIP-exo have become increasingly popular in genomic research. In general, ChIP-seq/-exo data analysis begins with filtering raw reads by their quality, mapping sequence reads to the reference genome, followed by peak visualization, peak calling, motif search, and/or other downstream analyses. Accordingly, specialized bioinformatic tools have been progressively developed to handle different types of data generated at each stage. These open-source tools are often available as standalone command-line programs, applications with graphical user interface (GUI) or web services. While there are computational pipelines to streamline ChIP-seq data analysis such as Cistrome [11]**,** ChIPseeqer [12], ChiLin [13]**,** and DROMPA [14], lack of similar efforts was made for ChIP-exo data processing.

Herein, we introduce ChIP-exo Analysis Pipeline (ChEAP), which incorporates major tools for NGS data analysis into a convenient script. From the raw sequencing files, ChEAP performs essential processing steps to generate output files for visualization of ChIP-exo signal. Dataset of RpoN sigma factor in *E. coli* K-12 MG1655 was provided to demonstrate how ChEAP could facilitate the workflow of ChIP-exo data analysis which ultimately, contributes to a further understanding of RpoN regulation in *E. coli*.

## 2 Materials and methods

### 2.1 ChEAP: ChIP-exo analysis pipeline

ChEAP was constructed with the integration of bioinformatic tools such as FASTX-Toolkit for trimming of poor quality reads (https://hannonlab.cshl.edu/fastx_toolkit/), Bowtie for reads mapping [15], and SAMtools for sorting and indexing alignments [16]. ChEAP was implemented on Google Colab – a free cloud computing platform, allowing researchers to execute and share Python code on Google’s cloud servers. Besides the standard libraries in Python, popular modules for NGS analysis such as Biopython [17], and Pysam were incorporated as part of this pipeline. Visualization of the resulting alignment was done via the MetaScope genome browser.

### 2.2 Peak calling

MACE – a peak calling tool optimized for ChIP-exo data [18], was used to perform candidate peak detection from biological replicates. The maximum distance for border pairing was set to 50 bp (-m 50). MACE outputs in BED format were converted to GFF files using an in-house script, enabling its results to be verified along with the ChIP-exo signals and the reference genome using MetaScope. Further manual curation to remove false-positive peaks was carried out via the MetaScope software. Genome-wide mapping of RpoN binding profile was visualized using Circos [19].

### 2.3 Motif discovery

From the peak calling outputs, sequences of RpoN binding site were obtained, and their lengths were extended to include 15 bp at both ends of the RpoN binding peak. The analysis of enriched RpoN binding motif was done via MEME suite [20]. Default settings were used except for the window search size that was set to 20 bp (-w 20) and the minimum number of motif occurrences that was set to at least 90 % of the input peaks.

### 2.4 Cluster of orthologous groups (COG) functional annotation

COG analysis was performed via UBLAST algorithm provided by the USEARCH tool [21]. The sequence information was based on the COG database of NCBI [22]. The functional category was confirmed through a local alignment with the custom database of COG orthologue information. Distribution of genes in each COG group was presented using the Seaborn library in Python [23].

## 3 Results

### 3.1 Implementation of ChEAP

ChEAP workflow (Fig. 1B) begins with the simple installation of required packages, importing of essential modules, and uploading of files into the Colab notebook. Reference genome (GenBank format) serves as the basis for ChEAP to extract the annotation data and create a FASTA file for subsequent building of the index by Bowtie. If the sample provided is a paired-end sequencing dataset, information obtained from read_1 alone is sufficient to detect protein binding locations. In ChIP-exo, the 5′ end of read_1 is defined by the λ exonuclease digestion. Thus, read_1 sequence of forward and reverse strands indicate the left and right borders of TF binding site, respectively. Reads of low quality (Phred score below 33) are filtered out, and trimmed down to the remaining length of 31 bp using FASTX-Toolkit. Bowtie is incorporated to map sequencing reads to the indexed reference genome. The resulting alignment in SAM format can be converted to 1) sorted BAM files using standard SAMtools commands (view, sort, index) and 2) GFF files using the custom functions provided. These functions iterate through each mapped read in SAM files and calculate the read coverage per base location in the genome.

**Fig. 2:**
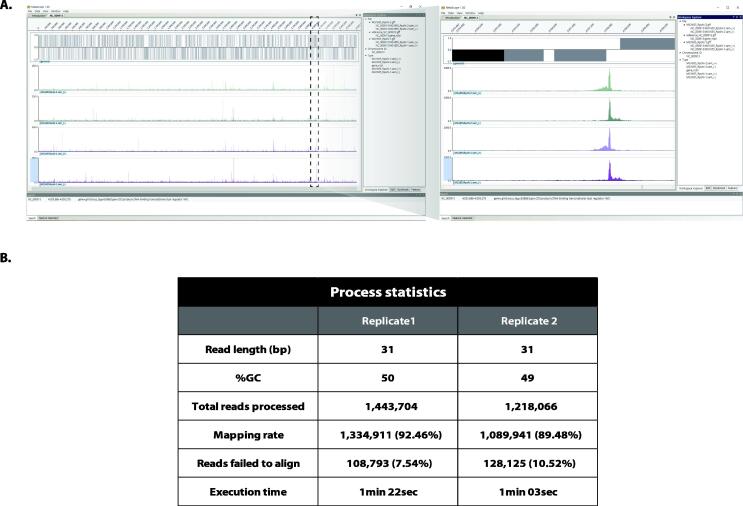
Statistics and visualization of ChIP-exo dataset with ChEAP. (A) Screenshot of MetaScope view. The genome-wide signal distribution from ChIP-exo replicates of RpoN in *E. coli* K-12 MG1655 can be compared with the genome annotation (left). Signal of individual RpoN binding peak can be further examined by zooming-in (right). (B) Table of processing statistics of ChIP-exo RpoN in *E. coli* K-12 MG1655.

The GFF outputs of genome annotation and the alignment data can be imported into the MetaScope software. MetaScope provides a visual inspection of read distribution thus, allows users to intuitively assess and analyze the obtained data. Results from multiple genome-scale experiments can be viewed in one window, along with the genomic annotation (Fig. 2A). Thus, direct comparative analysis between each experiment is possible. Editing features, panning, zooming, and reordering of the data tracks enable high-quality data curation as we consider not only the numerical value but also the shape of each signal peak. Additional track operations such as differencing, summing, filtering adjusting, and assigning ID, which prove to be helpful for NGS data analysis, are included. MetaScope runs on the Windows operating system (OS) that most users are familiar with. Hence, downloading and launch of the application are straightforward.

The pipeline generates alignment results in both BAM and GFF formats. BAM files could be used as the input for further downstream analysis such as peak calling. ChEAP allows flexible modifications of pipeline parameters. For instance, users may adjust the quality cutoff and optimal trim length of their reads. ChIP-exo data of different TFs from multiple species can be analyzed simultaneously. ChEAP was written in Python with its applicability leveraged in the Google Colab environment. The pipeline generates, and stores output files into structured directories found in the user’s Google Drive account with no settings of file paths required. The computing resources allocated by free-tier Google Colab support the basic requirement for an easy start of analysis. For users who prefer using local computing power for large datasets, ChEAP can be downloaded as a IPYNB or a PY file from GitHub. Alternatively, ChEAP on Colab can be connected directly to the local runtime using Jupyter and its extension, jupyter_http_over_ws.

### 3.2 ChEAP result for *E. Coli* RpoN ChIP-exo dataset

**Fig. 3:**
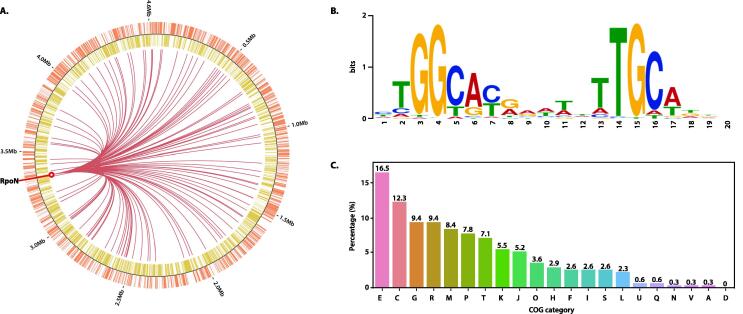
Reconstruction of *E. coli* RpoN protein-DNA interactions with ChEAP results at the genome scale (A) Circular representation of genome-wide binding landscape of *E. coli* RpoN. The outermost circle (orange) and inner circle (yellow) denote forward and reverse strands of the genome annotation, respectively. Dark red lines indicate 113 binding sites of *E. coli* RpoN. (B) Sequence motif of *E. coli* RpoN binding sites is calculated with MEME [20]. (C) Functional classification of genes associated with RpoN bindings is calculated. Each COG category is represented by its one-letter code. [E] Amino acid transport & metabolism, [C] Energy production & conversion, [G] Carbohydrate transport & metabolism, [R] General function prediction only, [M] Cell wall/membrane/envelope biogenesis, [P] Inorganic ion transport & metabolism, [T] Signal transduction mechanisms, [K] Transcription, [J] Translation/ribosomal structure & biogenesis, [O] Post-translational modification/protein turnover & chaperones, [H] Coenzyme transport & metabolism, [F] Nucleotide transport & metabolism, [I] Lipid transport & metabolism, [S] Function unknown, [L] Replication/recombination & repair, [U] Intracellular trafficking/secretion & vesicular transport, [Q] Secondary metabolites biosynthesis/transport & catabolism, [N] Cell motility, [V] Defense mechanisms, [A] RNA processing and modification, [D] Cell cycle control/cell division/chromosome partitioning.

The RpoN ChIP-exo dataset we used to demonstrate ChEAP application was obtained using the anti-*E. coli* RpoN antibodies whose specificity was verified (data not shown). The quality statistics of RpoN ChIP-exo replicates including their mapping rates, the number of reads processed, and execution time for each sample were summarized in Fig. 2B. ChEAP runtime was 1 min 22 s and 1 min 03 s to process samples of 1,443,704 and 1,218,066 reads, respectively. Because the reference index needed to be built only once, subsequent sequencing data mapping against the same index will be even faster. In the downstream analysis, peak calling using MACE and its post-curation outputs revealed 113 putative RpoN binding sites (Table S1) with their genome-wide binding landscape depicted in Fig. 3A. Of which, the consensus sequence motif of ntGGcacgnntntTGCann (n = uncharacterized) (Fig. 3B) was detected in 102 peaks.

It was established that RpoN reversibly associates with the core RNA polymerase (RNAP) to recognize promoters with the consensus sequences at −12 and −24 bp relative to the transcription start site (TSS), and transcription initiation is dependent on interaction with a member of the bacterial enhancer-binding proteins (bEBPs) family [24]. In this study, the motif with highly conserved GG at the −24 region, and GC at the −12 region resembles the RpoN binding motif that was previously characterized by ChIP-seq and microarray [3], [25]. As ChIP-exo and ChIP-seq techniques provide high resolution data, it is possible to distinguish between intergenic and intragenic binding sites. Instances of intragenic RpoN bindings were reported in several bacterial species [26], [27]. In *E. coli*, RpoN regulation has been investigated by ChIP-chip and ChIP-seq method [2], [3]. There were 60 ChIP-exo peaks in our list overlapping with those from ChIP-seq and ChIP-chip studies. Of these, 44 and 16 peaks were labeled as intergenic and intragenic respectively in our dataset (Supplementary Figure S1A). Bonacora et al., 2015 reported a total of 135 high stringency ChIP-seq peaks [3]. Of which, more than half of them (85/135) were found to be intragenic. Likewise, our ChIP-exo dataset suggested that more than half of the RpoN binding locations (53.1 %) were found within genes (Supplementary Table S1). Further analyses revealed that 58 % (29/50) of the intergenic ChIP-seq peaks, and 58.8 % (50/85) of the intragenic ChIP-seq peaks overlapped with our ChIP-exo binding sites (Supplementary Figure S1B). The remaining 28/113 ChIP-exo peaks were unique to our experimental condition. The enriched RpoN motif was also observed when conducting motif search with these unique targets (Supplementary Figure S1C).

### 3.3 Reconstruction of *E. Coli* RpoN sigmulon and its functional analyses

From the RpoN binding locations, neighboring genes could be the potential targets regulated by RpoN sigma factor. A total of 294 target genes in 110 transcription units (TUs) were identified to be candidates of the RpoN sigmulon (Table S2). Of which, 108 genes were previously characterized to be RpoN sigmulon members as listed in EcoCyc [28] and RegulonDB [29], leaving 186 genes being newly identified.

Based on the COG protein database, 294 putative targets were classified into 20 different COG functional groups (Fig. 3C) including amino acid metabolism and transport (E), energy production and conversion (C), carbohydrate metabolism and transport (G), cell wall/membrane/envelope biogenesis (M), and inorganic ion transport and metabolism (P). The result indicated an apparent diversity of RpoN-dependent genes in which a high abundance of these genes (16.5 %) has their functions involved in amino acid transport and metabolism. This result is congruent with previous findings where RpoN has been identified for its role in nitrogen assimilation [24]. In addition, our COG analysis revealed that 12.3 % of the gene functions were related to energy production and conversion. As compared to the COG distribution of total genes in *E. coli* K-12 MG1655, putative RpoN sigmulon accounted for 6.84 % of the total CDS and 7.69 % of the total genes. In the E, C, M, T, and A categories, putative RpoN sigmulon contributed to more than 10 % of the total genes (Supplementary Figure S2).

## 4 Discussion

Due to the high volume of sequencing data, software for NGS analyses often depend on several resources, including storage space, computer units, network connection, compatible software environment, and local machine OS. In light of such challenges, cloud computing has provided an alternative solution to how data can be managed, stored, and processed. Colab with a dedicated storage, and a user-friendly layout of Jupyter notebooks for stepwise execution of codes has quickly become favorable. Colab allows users to process multiple files through shared scripts, without the need to install and configure additional software in different local environments. Furthermore, Colab operates independently from the local machine OS. Therefore, ChEAP implemented on this platform promotes its utility and access to large amounts of data via Google Drive or GitHub connection. Users could exploit the free computing resources allocated by Google Colab or connect the script to their local computing power for analysis of large datasets. While the pipeline can be installed locally, ChEAP on Colab can be connected to the local runtime and executed directly as is.

As demonstrated with the RpoN ChIP-exo data, ChEAP allows even beginners to perform the genome-wide binding site analysis easily and quickly. Various ChIP-exo data of an organism could be collectively processed via ChEAP, followed by visualization of multiple data tracks on MetaScope where overlap binding regions could be assessed. Additional information about the orientation of each binding site as well as their mode of regulation can be revealed by integrating it with data from other high-throughput experiments such as RNA-seq. MetaScope facilitates the visual comparison between ChEAP outputs and RNA-seq results obtained under the same experiment condition. It would allow us to observe if there are instances of pervasive promoters and anti-sense TSS associated with RpoN binding sites [3], [30].

In response to the environmental changes, bacteria render their adaptive ability by acquiring different sigma factors to regulate different set of regulons. In growing cells, most transcription is initiated with the housekeeping sigma factor group (sigma-70 class) and alternative sigma factors control a specific set of target regulons during certain stress conditions, growth transitions, and morphological changes [31]. Thus, comparative analysis to discover the presence of overlapping promoters or detect the possible competitive binding between sigma factors are often of great interest [32], [33]. Due to the fundamental differences in their sequences, promoter structures, and functions, RpoN constitutes a different family from the sigma-70 class group [34], [35]. Their modes of transcription initiation were found to have unique features. Unlike sigma-70 class group, RpoN must require a class of activator – bEBPs which hydrolyze ATP and form an interaction with RNAP-RpoN complex to initiate transcription [36]. This absolute requirement of bEBPs renders a tighter expression control as it allows the regulated genes to be completely suppressed if needed. This stringent regulation by RpoN is essential in modulating energy-consuming activities such as the nitrogen metabolism. Indeed, COG analysis of putative RpoN sigmulon identified in our ChIP-exo dataset revealed the presence of genes with function related to nitrogen metabolism. As compared to the COG distribution of total genes in *E. coli* K-12 MG1655, putative RpoN sigmulon contributed to more than 10 % in the E, C, M, T, and A categories. However, this comparison was only based on cell growth in standard M9 media with 0.2 % w/v glucose. Hence, the COG distribution may vary across different experimental conditions. Further comparisons with transcriptome data of both WT and knockout strains could reveal a more comprehensive set of RpoN regulated genes contributing to different COG categories. Previously, it was also suggested that for RpoN-dependent genes with functions unrelated to nitrogen assimilation, such control may prevent the catastrophic depletion of metabolites and energy resources or partially counteract the adverse conditions [25]. Additionally, sigma factors binding sites to orthologous genes could reveal their conserved or diverged regulatory patterns in different conditions. In particular, previous studies have shown different genome-wide binding patterns of RpoN depending on the nitrogen availability in the culture media (nitrogen-limiting or nitrogen-repleting condition) [2], [3]. A comparison between our ChIP-exo data (RpoN data in *E. coli* K-12 MG1655) and a recent study (RpoN data obtained by gSELEX in *E. coli* K-12 W3110) [37] showed an interesting number of conserved DNA binding targets (Supplementary Fig. 3). Therefore, further investigation into the uncharacterized RpoN targets under various conditions might provide interesting insights of RpoN regulation.

Collectively, ChEAP enables researchers to process multiple ChIP-exo datasets of different DNA-binding proteins simultaneously. ChEAP alleviates the bottleneck of ChIP-exo data analysis and facilitates the comparative study of different regulatory DNA-binding proteins.

## 5 Conclusions

Leveraging on the strength of many popular tools, ChEAP provides a simple yet effective approach to manage several steps in ChIP-exo data analysis. In addition, ChEAP takes advantage of cloud-computing which facilitates sharing of codes and collaboration among researchers. This pipeline not only runs independently of the local operating systems but also processes multiple raw FASTQ files in a single run. This paper demonstrated the utility of ChEAP with the analysis of RpoN sigma factor to delineate its regulatory network in *E. coli*. Moreover, as ChEAP design emphasizes on flexibility and extensibility, this pipeline could certainly be developed as part of an extensive workflow or be optimized for analyzing ChIP-exo data of different organisms.

## CRediT authorship contribution statement

**Ina Bang:** Software, Methodology, Investigation, Data curation, Visualization, Writing – original draft, Writing – review & editing. **Linh Khanh Nong:** Investigation, Data curation, Writing – original draft, Writing – review & editing. **Joon Young Park:** Visualization, Writing – review & editing. **Hoa Thi Le:** Writing – review & editing. **Sang- Mok Lee:** Writing – review & editing. **Donghyuk Kim:** Conceptualization, Software, Methodology, Resources, Writing – review & editing, Project administration, Supervision, Funding acquisition.

## Declaration of Competing Interest

The authors declare that they have no known competing financial interests or personal relationships that could have appeared to influence the work reported in this paper.

## Data Availability

ChEAP implemented on the Google Colab platform is publicly available at [https://github.com/SBML-Kimlab/ChEAP]. The visualization software “MetaScope” is available at [https://github.com/SBML-Kimlab/MetaScope]. Raw data of ChIP-exo experiment have been deposited in the Gene Expression Omnibus under the accession code GSM1326363, GSM1326364.
