# Supplementary material for: ChEAP: ChIP-exo analysis pipeline and the investigation of Escherichia coli RpoN protein-DNA interactions
Source: Comput Struct Biotechnol J. 2022 Dec 2;21:99–104. doi: 10.1016/j.csbj.2022.11.053 (PMC9735260; doi:10.1016/j.csbj.2022.11.053)
Supplement: Supplementary data 1 [file mmc1.docx]

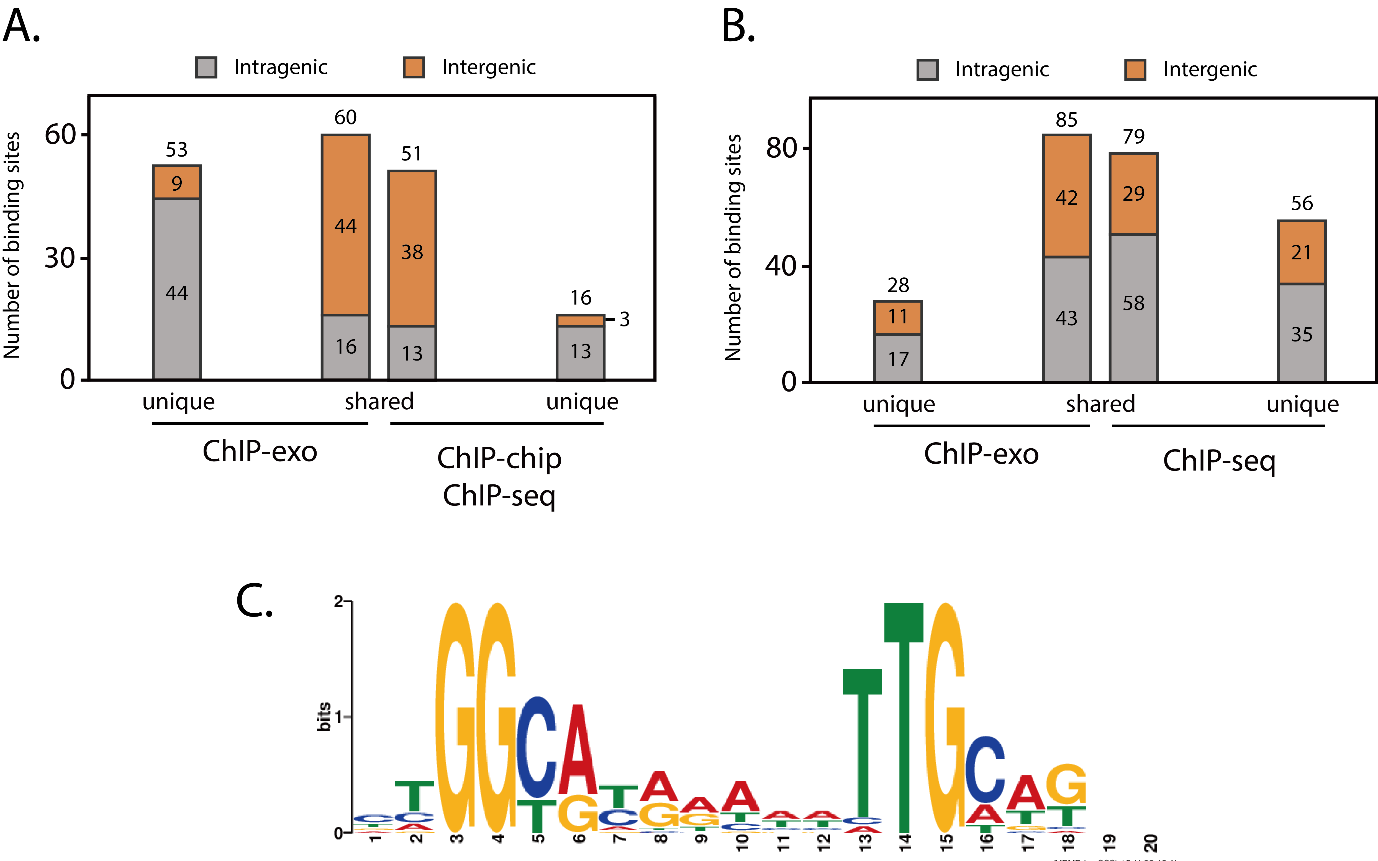


**Figure S1.** Statistical comparison of RpoN ChIP-exo binding sites with previously reported data. **(A)** Shared and unique targets identified by ChIP-exo, ChIP-chip [2], and ChIP-seq [3]. **(B)** Shared and unique targets identified by ChIP-exo and ChIP-seq [3]. **(C)** Consensus motif derived from 28 RpoN ChIP-exo peaks unique to this study.


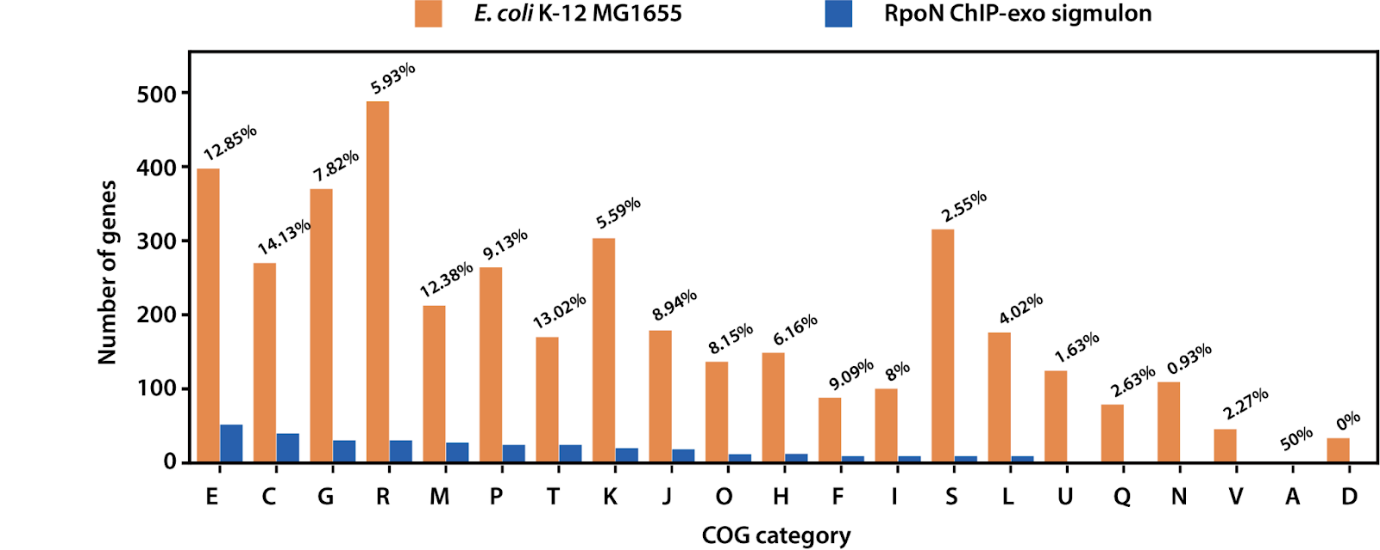

**Figure S2.** COG distribution of putative RpoN sigmulon and total genes in *E. coli* K-12 MG1655. Percentages indicated the ratio of putative RpoN sigmulon against the total number of genes in each COG category.

**
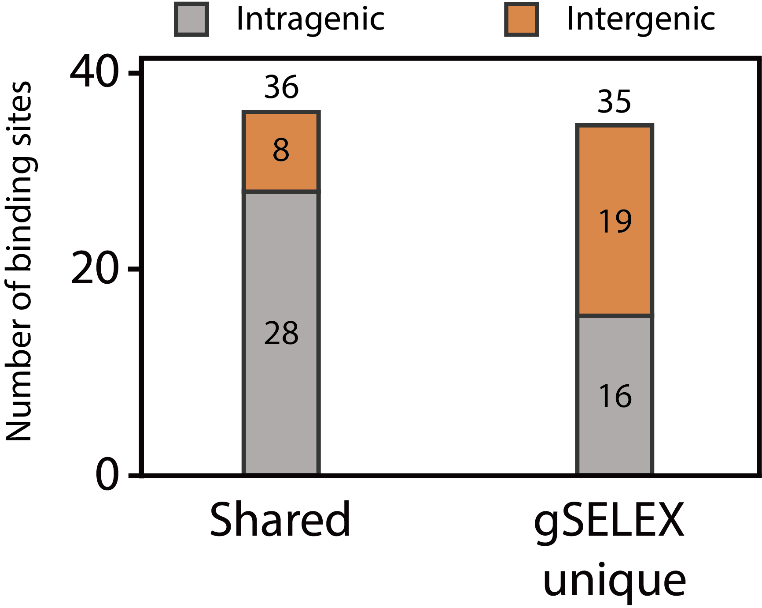
Figure S3. Shared and unique targets at the orthologous genes in *E. coli* K-12 MG1655 (ChIP-exo in nitrogen replete condition) and *E. coli* K-12 W3110 (gSELEX in nitrogen deplete condition) [37].** Shimada et al. reported a total of 71 RpoN holoenzyme binding sites in *E. coli* K-12 W3110 using the gSELEX method. Of these, we detected 36 RpoN binding sites close to their orthologs in MG1655, leaving 35 targets specific to W3110.
